# Adiponectin, in contrast to leptin, is not associated with body mass index, waist circumference and HOMA‐IR in subjects of a west‐African population

**DOI:** 10.14814/phy2.13718

**Published:** 2018-06-10

**Authors:** Bonaventure Awede, Diane Adovoekpe, Grace Adehan, Niall G. MacFarlane, Simon Azonbakin, Emmanuel Dossou, Marcellin Amoussou‐Guenou, François Djrolo

**Affiliations:** ^1^ Unité de Physiologie Faculté des Sciences de la Santé (FSS) Université d'Abomey‐Calavi Cotonou Benin; ^2^ Unité de Biophysique et de Médecine Nucléaire Faculté des Sciences de la Santé Université d'Abomey‐Calavi Cotonou Benin; ^3^ School of Life Sciences College of Medical Veterinary and Life Sciences University of Glasgow Glasgow United Kingdom; ^4^ Unité de Biologie Humaine Faculté des Sciences de la Santé Université d'Abomey‐Calavi Cotonou Benin; ^5^ Unité d'Endocrinologie et de Nutrition Faculté des Sciences de la Santé Université d'Abomey‐Calavi Cotonou Benin

**Keywords:** Adiponectin, insulin resistance, leptin, obesity

## Abstract

Factors associated with plasma levels of adiponectin and leptin were studied in adult subjects without diabetes from Cotonou in Benin (West‐Africa). Seventy (70) men and 45 women were included in the study. Anthropometric variables were measured and a venous blood sample was drawn from each subject, after an overnight fasting period, for measurement of plasma glucose, insulin, leptin, and adiponectin levels. HOMA‐IR was determined to assess insulin resistance. Adiponectin and leptin levels were higher in women than in men (with adiponectin 18.48 ± 12.77 vs.7.8 ± 10.39 *μ*g/mL,* P *<* *0.0001, and leptin 30.77 ± 19.16 vs. 8.66 ± 8.24 ng/mL,* P *<* *0.0001). Fasting insulin level and HOMA‐IR were also higher in the females. Hyperleptinemia was observed in 66,96% of subjects and hypoadiponectinemia was present in 44.35% of subjects. In both men and women, leptin correlated with age (*r* = 0.2; *P* = 0.02), BMI (*r* = 0.572; *P* < 0.0001), waist circumference (*r* = 0.534; *P* < 0.0001), fasting insulin (*r* = 0.461; *P* < 0.001), and HOMA‐IR (*r* = 0.430; *P* < 0.0001). No significant correlation was observed for adiponectin levels with these variables. Only in women, adiponectin was inversely correlated with fasting glucose (*r* = −0.423; *P* < 0.004). These data confirm previous descriptions of leptin but suggest that variations in factors determining serum adiponectin levels observed between ethnicities could also been seen between populations from the same ethnicity.

## Introduction

Adipose tissue, in addition to its basic role for fat storage, functions as an endocrine organ to produce a range of hormones called adipokines or adipocytokines among which are adiponectin and leptin (Ahima 2006). The adipokines regulate energy homeostasis, immunity function and reproduction and provide feedback on the state of the bodies energy reserves. Any significant change in energy reserve will alter adipokines secretion and disturb their influence on the control of body functions. This is particularly relevant with the dramatic increase in overweight and obese individuals across the world as obesity is an important risk factor for metabolic and cardiovascular diseases.

Many studies have been dedicated to the study of factors determining or associated with plasma levels of leptin and adiponectin in several populations. Correlations between these plasma levels and gender, age, body mass index (BMI), abdominal obesity, adipose fat mass have been reported (Ruige et al. 1999; Monti et al. 2006; Zuo et al. 2013). However, several studies, generally carried out in western countries, have reported significant differences in the profile of these adipokines between the main ethnic groups (Caucasians, African Americans, Asian, Hispanic) which could partly explain differences in risk for cardiovascular diseases (Mente et al. 2010; Khan et al. 2012; Rasmussen‐Torvik et al. 2012; Azrad et al. 2013; Morimoto et al. 2014).

In sub‐Saharan Africa, only a few studies considered adiponectin and leptin profiles with the correlation between leptin and body mass index (BMI) or waist circumference confirmed in South African and Cameroonian subjects (Ntyintyane et al. 2009; Ayina et al. 2016). Similarly, Cameroonian, South African and West African populations show negative correlations between adiponectin and BMI, waist circumference, triglycerides levels, and HOMA‐IR (Schutte et al. 2007; Meilleur et al. 2010; Ayina et al. 2016), however, such correlations are not found in black South African subjects with coronary artery disease (Ntyintyane et al. 2009).

Thus, the factors influencing adipokine regulation might vary between sub‐Saharan populations due to their economic, genetic and social diversity. Furthermore, while West Africa has been dominated by programs that have addressed undernutrition, the region is now undergoing a significant transition where urbanization and technological development is leading to increasing rate of obesity. The aim of the current study is to determine whether changes in adiponectin and leptin may be relevant to consider the pathophysiology of obesity‐related diseases that are rapidly increasing in Benin.

## Participants and Methods

Study participants were adult, without diabetes, from Cotonou in the Republic of Benin, West Africa. They were recruited during July–August 2015 and provided written informed consent form prior to participation. The study was approved by the Ethics Committee of the Institute of Advanced Biomedical Sciences, Cotonou (CER‐ISBA) and was conducted in accordance with Helsinki Declaration.

### Data collection

Participants fasted prior to data collection (from 2000 h to 0800 h). In the morning, information on the participants medical history and lifestyle was collected. Anthropometric measurements (height, weight, and waist circumference) and blood pressure were recorded and then BMI determined. A venous blood sample was drawn to allow subsequent measurement of blood glucose and hormones (adiponectin, insulin, and leptin).

### Analytic methods

Blood glucose levels were measured by glucose oxidase method on plasma obtained after centrifugation. Plasma insulin, leptin, and adiponectin levels were measured by radioimmunoassay (RIA) using specific kits according to the manufacturer instructions. Insulin IRMA, Leptin (human) and adiponectin (human) kits were purchased from Demeditec Diagnostic (Germany).

### Insulin resistance index

Insulin resistance (HOMA‐IR) was assessed by a homeostatic model using fasting plasma glucose (mmol/L) and fasting plasma insulin (*μ*UI/mL) as follows:

HOMA-IR=[fasting plasma glucose]×[fasting plasma insulin]/22.5

### Statistical analysis

Data were expressed as means ± SD or as proportions. Comparison of proportions between groups was made by Chi‐square and that of means made by ANOVA or by Student *t* test (significance was set at *P* < 5%). Spearman correlation coefficients were used to evaluate the association between adiponectin and leptin levels with other variables.

## Results

One hundred twenty‐four subjects were recruited for the study. Five subjects with high fasting blood glucose levels (blood glucose > 1.1 g/L) and four subjects with outlier values of serum leptin, adiponectin, or insulin, were excluded from the final study. Thus, 115 subjects (70 men and 45 women) were included in the analysis.

### Characteristics of study subjects

The main anthropometric and metabolic characteristics of subjects are presented in Table 1.

**Table 1:** Clinical and metabolic characteristics of subjects

Variables	All subjects (*n* = 115)	Male (*n* = 70)	Female (*n* = 45)	*P*‐value
Age (years)	35.9 ± 11.8	36.4 ± 11.6	35.1 ± 12.2	0.57
Body mass (kg)	71.82 ± 15.19	75.34 ± 13.45	66.36 ± 16.34	0.002
BMI (kg/m^2^)	25.73 ± 5.48	25.22 ± 4.39	26.53 ± 6.82	0.213
Waist circumference (cm)	88.20 ± 13.00	88.60 ± 11.80	87.5 ± 14.5	0.64
Fasting glucose (g/L)	0.85 ± 0.09	0.87 ± 0.1	0.82 ± 0.07	0.00123
Fasting insulin (*μ*U/mL)	4.98 ± 3.14	4.09 ± 2.54	6.36 ± 3.51	0.00011
HOMA	1.05 ± 0.69	0.90 ± 0.62	1.3 ± 0.75	0.00224
Leptin (ng/mL)	17.31 ± 17.33	8.66 ± 8.24	30.77 ± 19.16	<0.0001
Adiponectin (*μ*g/mL)	11.98 ± 12.48	7.8 ± 10.39	18.48 ± 12.77	<0.0001

Values are means ± standard deviation; *P*‐value refers to comparison between male and female subjects.

There was no significant difference in age between men and women. Body mass was higher in men than in women but BMI and waist circumference were similar in both groups. However, based on BMI, 28.89% of women and 12.85% of men were obese whereas the proportion of overweight participants was higher in men (38.57%) than women (20%). The proportion of overweight and obese subjects increased with age, with 12% and 4% of study participants overweight and obese in those less than 25 years of age, increasing to 33.93% and 19.64% in those aged 25–44 years and increasing further to 41.18 and 29.41% in those more than 44 years of age.

Fasting blood glucose was in normal range in all subjects but was slightly higher in males compared to females (0.87 ± 0.10 vs. 0.82 ± 0.07 g/L, *P *<* *0.01). Fasting insulin and HOMA‐IR was higher in females than males (6.36 ± 3.51 vs. 4.09 ± 2.54 *μ*U/mL, *P *<* *0.01 and 1.3 ± 0.75 vs. 0.90 ±* *0.62 AU, *P *<* *0.01, respectively). Adiponectin and leptin were much higher in females (adiponectin was 18.48 ± 12.77 vs. 7.8 ± 10.39 *μ*g/mL, *P *<* *0.0001 and leptin was 30.77 ± 19.16 vs. 8.66 ± 8.24 ng/mL, *P *<* *0.0001). Hyperleptinemia ( i.e.where leptin is >5.6 ng/mL in males or >11.1 ng/mL in females) was observed in 66.96% of the participants and hypoadiponectinemia ( i.e.where adiponectin is <5 *μ*g/mL) was observed in 44.35% of the participants.

#### Leptin and age

Leptin was age dependent in both men and women. In men, leptin was 3.56 ± 3.63 ng/mL in those less than 25 years of age, 10.47 ± 9.90 ng/mL in those aged 25–44 years and 8.15 ± 4.57 ng/mL in those more than 44 years of age. In women, there was a progressive increase in leptin from 20.26 ± 13.09 ng/mL in those less than 25 years of age to 30.60 ± 16.20 ng/mL in those aged 25–44 years and to 40.07 ± 22.61 ng/mL in those more than 44 years of age.

#### Leptin and BMI

In men and in women, a progressive increase in leptin was observed as BMI increased. In men leptin was 1.88 ± 0.76 ng/mL in those with low BMI, 5.09 ± 4.57 ng/mL in those with normal BMI, 9.70 ± 4.14 ng/mL in those where the BMI indicated being overweight, and 20.41 ± 14.99 ng/mL in those where the BMI indicated obesity. In females, leptin was 16.34 ± 15.52 ng/mL in those with low BMI, 19.19 ± 10.71 ng/mL in those with normal BMI, 35.25 ± 16.13 ng/mL in those where the BMI indicated being overweight, and 48.82 ± 17.45 ng/mL in those where the BMI indicated obesity.

#### Leptin and waist circumference

Leptin was much higher in participants with high waist circumference (>94 cm in men and >80 cm in women). In men, leptin was 5.63 ± 4.24 ng/mL in participants with normal waist circumference and 14.6 ± 10.26 ng/mL in those with a high waist circumference. In women, leptin was 14.68 ± 8.67 ng/mL in participants with normal waist circumference and 39.65 ± 17.51 ng/mL in those with a high waist circumference.

#### Leptin and fasting insulin

An increase in leptin with insulin was observed in women. Leptin was 23.95 ± 17.51 ng/mL in those with insulin below the normal range, 31.15 ± 17.20 ng/mL in those whose insulin was within the normal range, and 63.88 ± 7.40 ng/mL in those with hyperinsulinemia.

#### Leptin relationship with age, BMI, waist circumference, fasting insulin, and HOMA‐IR

The relationship between leptin with age, BMI, waist circumference, fasting insulin, and HOMA‐IR were considered by Spearman correlation coefficient and the results are presented in Table 2.

**Table 2:** Correlation between leptin and adiponectin levels and anthropometric and biological variables in all subjects (males and females)

	Leptin		Adiponectin	
	*r*	*P*	*r*	*P*
Age	0.2	0.02	0.1	0.101
BMI	0.572	<0.0001	−0.001	0.996
Waist circumference	0.534	<0.0001	−0.011	0.904
Fasting insulin	0.461	<0.0001	0.155	0.099
Fasting glucose	−0.129	0.161	−0.309	0.001
HOMA‐IR	0.430	<0.0001	0.114	0.224

*r*, Spearman correlation coefficient; *P*:* P*‐value, *n* = 115.

In summary, there was a positive association between leptin and age (*r* = 0.2; *P* = 0.02), BMI (*r* = 0.572; *P* < 0.0001), waist circumference (*r* = 0.534; *P* < 0.0001), fasting insulin (*r* = 0.461; *P* < 0.0001), and HOMA‐IR (0.430; *P* < 0.0001) but no association with fasting glucose. When considered in relation to sex, as presented in Tables 3 and 4, leptin correlated with the same variables in both men and women.

**Table 3:** Correlation between leptin and adiponectin levels and anthropometric and biological variables in men

	Leptin		Adiponectin	
	*r*	*P*	*r*	*P*
Age	0.398	0.001	0.282	0.18
BMI	0.682	<0.0001	−0.130	0.283
Waist circumference	0.810	<0.0001	0.006	0.961
Fasting Insulin	0.280	0.019	−0.130	0.283
Fasting glucose	0.215	0.074	−0.126	0.300
HOMA‐IR	0.296	0.013	−0.102	0.403

*r*, Spearman correlation coefficient; *P*:* P*‐value, *n* = 70.

**Table 4:** Correlation between leptin and adiponectin levels and anthropometric and biological variables in women

	Leptin		Adiponectin	
	*r*	*P*	*r*	*P*
Age	0.357	0.016	−0.073	0.633
BMI	0.739	<0.0001	−0.060	0.696
Waist circumference	0.779	<0.0001	−0.068	0.658
Fasting Insulin	0.372	0.012	0.119	0.436
Fasting glucose	−0.114	0.545	−0.423	0.004
HOMA‐IR	0.345	0.020	0.036	0.812

*r*, Spearman correlation coefficient; *P*:* P*‐value, *n* = 45.

#### Adiponectin and age

In men, there was a trend to an increase in adiponectin when age increased. Adiponectin level was thus 4.80 ± 5.97 *μ*g/mL in those less than 25 years of age, 6.28 ± 8.16 *μ*g/mL in those aged 25–44 years, and 12.8 ± 14.63 *μ*g/mL in those more than 44 years of age. In women, however, no difference in adiponectin was observed with age.

#### Adiponectin and BMI

In men, adiponectin level was 15.62 ± 11.12 *μ*g/mL in those with low BMI, 6.41 ± 8.75 *μ*g/mL in those with normal BMI, 8.76 ± 12.49 *μ*g/mL in those where the BMI indicated being overweight, and 6.09 ± 7.45 *μ*g/mL in those where the BMI indicated obesity. In women, adiponectin level in those where the BMI indicated being overweight (28.39 ± 13.14 *μ*g/mL) tended to be higher than adiponectin in those with low BMI (19.74 ± 15.21 *μ*g/mL), in those with normal BMI (16.00 ± 11.60 *μ*g/mL), and in those where the BMI indicated obesity (15.13 ± 11.59 *μ*g/mL).

#### Adiponectin and waist circumference

There was no effect of waist circumference on adiponectin. In men, adiponectin was 6.28 ± 8.54 *μ*g/mL in participants with normal waist circumference and 10.54 ± 12.82 *μ*g/mL in participants with high waist circumference. In women, adiponectin was 17.35 ± 12.48 *μ*g/mL in participants with normal waist circumference and 19.10 ± 13.10 *μ*g/mL in participants with high waist circumference.

#### Adiponectin and fasting insulin

In men a trend to a decrease in adiponectin when insulin increased was observed. Adiponectin was 9.51 ± 12.08 *μ*g/mL in those with insulin below the normal range, 5.21 ± 6.24 *μ*g/mL in those whose insulin was within the normal range and 1.43 in the only participant with hyperinsulinemia. In women, no difference in adiponectin related to insulin was observed.

#### Adiponectin relationship with age, BMI, waist circumference, fasting insulin, and HOMA‐IR

The relationship between adiponectin with age, BMI, waist circumference, fasting insulin, and HOMA‐IR were considered by Spearman correlation coefficient and the results are presented in Table 2.

In summary, there was no association between adiponectin and age, BMI, waist circumference, fasting insulin, HOMA‐IR and but adiponectin was inversely related to fasting glucose. When considered in relation to sex, adiponectin lost any relationship with fasting glucose in men. In women, the inverse relationship between adiponectin and fasting glucose persisted.

## Discussion

In the present study serum adiponectin and leptin have been measured and their relationships with gender, age, BMI, waist circumference, fasting insulin, and insulin resistance have been considered in a West African population. Although previous studies in West Africans have been reported (Meilleur et al. 2010; Oshodi et al. 2012), this study considers these adipokines in participants from an area that has been subjected to nutritional transitioning due to urbanization in the Republic of Benin.

Adiponectin and leptin concentrations were observed to be higher in females within this population. These data are consistent with data previously reported on either sub‐Saharan and African American subjects and for data obtained in studying other ethnicities (Ruige et al. 1999; Meilleur et al. 2010; Oshodi et al. 2012; Bidulescu et al. 2013; Zuo et al. 2013; Ayina et al. 2016). The sex difference is thought to result, especially for leptin, from many mechanisms including sex steroid hormone action (stimulating effect of oestrogens and inhibitory effect of androgen on leptin expression) and an increased expression of leptin in subcutaneous adipose tissue compared to visceral adipose tissue (Park and Ahima 2014). The menstrual status of women could have helped to appreciate the influence of sex steroid hormones on leptin level, but these data were not available.

The proportion of participants with hyperleptinemia or hypoadiponectinemia was high in this study. These data suggest that a large proportion of subjects of this population probably present a reduced insulin sensitivity and that they are at increased risk for developing metabolic and cardiovascular diseases.

A direct relationship was observed between leptin and BMI, waist circumference, age, fasting insulin, and HOMA‐IR. These data are consistent with the majority of previous studies conducted on African Americans, sub‐Saharan and other ethnicities (Ruige et al. 1999; Monti et al. 2006; Ntyintyane et al. 2009; Zuo et al. 2013; Ayina et al. 2016). These data suggest that the factors influencing leptin may be similar across ethnicities but that leptin concentrations may differ. In contrast, no adiponectin relationship with BMI, waist circumference, fasting insulin, and insulin resistance was observed. While these data conflict with some of the data previously reported for studies in Caucasians, African Americans, and sub‐Saharans (Schutte et al. 2007; Meilleur et al. 2010; Bidulescu et al. 2013; Ayina et al. 2016), they are consistent with other published data.

In a comparative study on African American and Caucasian women, the inverse relationship between adiponectin with BMI, fasting insulin, and HOMA‐IR was only observed in the Caucasians (Hulver et al. 2004). Furthermore, no relationship between adiponectin and BMI, waist circumference, and insulin resistance has been previously described in Asian populations (Snehalatha et al. 2008; Mente et al. 2010) with similar results in an Egyptian population (Elokely et al. 2010).

The variability in data makes consideration of any putative association between adiponectin with BMI, waist circumference, and insulin resistance difficult. Moreover, a meta‐analysis of published data suggest that there is no relationship between body mass index and adiponectin in healthy subjects (Kuo and Halpern 2011). In our study, only BMI and Waist circumference have been used as measure of adiposity and since ethnic difference in fat distribution have been described previously this might limit consideration of plasma adiponectin levels in this population. However, the data are restricted to participants from an urbanised area of West Africa and may allow us to consider whether the observed differences reflect economic and social transition.

## Conclusion

Our study showed that factors associated to leptin levels in sub‐Saharan subjects are similar to those described in populations of other ethnicities. In contrast to leptin and in addition to ethnic differences previously reported, our data suggest that factors influencing adiponectin regulation vary even between populations of sub‐Saharan region. Further studies, in each population group, will thus be useful to determine these factors for a better understanding of the involvement of adiponectin in cardiovascular and metabolic diseases.

## Conflict of Interest

The authors have no conflict of interest to declare.
